# Real-Time Adaptive Nanofluid-Based Lubrication in Stainless Steel Turning Using an Intelligent Auto-Tuned MQL System

**DOI:** 10.3390/ma18204714

**Published:** 2025-10-14

**Authors:** Mahip Singh, Amit Rai Dixit, Anuj Kumar Sharma, Akash Nag, Sergej Hloch

**Affiliations:** 1 Innovation Hub UP, Dr. APJ Abdul Kalam Technical University, Lucknow 226031, India; mahipsingh91@gmail.com (M.S.); anujksharma@cas.res.in (A.K.S.); 2 Department of Mechanical Engineering, Indian Institute of Technology (ISM), Dhanbad 826004, India; amitraidixit@iitism.ac.in; 3 Centre for Advanced Studies, Dr. APJ Abdul Kalam Technical University, Lucknow 226031, India; 4 Faculty of Mechanical Engineering, VSB—Technical University of Ostrava, Poruba, 708 00 Ostrava, Czech Republic; akash.nag@vsb.cz; 5 Faculty of Manufacturing Technologies, TUKE with a Seat in Prešov, 080 01 Prešov, Slovakia

**Keywords:** auto-tuned MQL, surface roughness, cutting force, adaptive machining

## Abstract

Achieving optimal lubrication during machining processes, particularly turning of stainless steel, remains a significant challenge due to dynamic variations in cutting conditions that affect tool life, surface quality, and environmental impact. Conventional Minimum Quantity Lubrication (MQL) systems provide fixed flow rates and often fail to adapt to changing process parameters, limiting their effectiveness under fluctuating thermal and mechanical loads. To address these limitations, this study proposes an ambient-aware adaptive Auto-Tuned MQL (ATM) system that intelligently controls both nanofluid concentration and lubricant flow rate in real time. The system employs embedded sensors to monitor cutting zone temperature, surface roughness, and ambient conditions, linked through a feedback-driven control algorithm designed to optimize lubrication delivery dynamically. A Taguchi L9 design was used for experimental validation on AISI 304 stainless steel turning, investigating feed rate, cutting speed, and nanofluid concentration. Results demonstrate that the ATM system substantially improves machining outcomes, reducing surface roughness by more than 50% and cutting force by approximately 20% compared to conventional MQL. Regression models achieved high predictive accuracy, with R-squared values exceeding 99%, and surface analyses confirmed reduced adhesion and wear under adaptive lubrication. The proposed system offers a robust approach to enhancing machining performance and sustainability through intelligent, real-time lubrication control.

## 1. Introduction

In advanced manufacturing, the persistent challenge of achieving optimal lubrication during machining processes, specifically turning, has become a focal point for both productivity and sustainability. Though effective at dissipating heat and reducing friction, traditional flood lubrication systems are increasingly criticized for their excessive fluid consumption, generation of hazardous waste, and environmental footprint [1,2]. As a result, the industry has shifted its focus to minimum quantity lubrication (MQL) systems, which apply a precisely controlled mist of lubricant directly to the cutting zone. This approach significantly reduces fluid consumption while also lowering the environmental footprint and occupational health risks associated with metal cutting [3,4,5]. However, as machining operations grow more complex and expectations regarding surface integrity, tool life, and process stability rise, it becomes clear that standard MQL lacks the responsiveness necessary for truly optimal, adaptive lubrication, especially under fluctuating process loads and varying thermal environments [6,7]. This forms the primary motivation behind the present work: to develop an adaptive lubrication paradigm, moving far beyond conventional preset or basic feedback designs.

A review of existing literature demonstrates the gradual evolution of lubrication control paradigms. Most conventional MQL systems deliver lubricant at a constant rate, tuned only in advance or via manual intervention. Such static designs, although resource-efficient, cannot respond dynamically to changing tool–workpiece interactions [8,9]. Modern adaptive MQL systems represent a key advance; they typically fall into one of several categories. Preset adaptive systems rely on lookup tables or scheduled fluid flow variations in response to expected or programmed changes in speed, feed, or preset temperature thresholds [10,11]. These are inherently limited by their lack of real-time insight, often failing when unexpected load spikes, wear progression, or sudden temperature shifts occur. More recent implementations integrate simple feedback mechanisms such as on–off or proportional controllers using signals from a single process indicator (most commonly temperature) to modulate fluid output. While this is a step towards absolute adaptability, single-parameter focus frequently results in over-lubrication or insufficient lubrication for certain machining states [12]. An additional problem is their narrow control horizon; they seldom, if ever, address the multifactorial nature of machining where tool wear, chip evacuation, micro-climate, and fluid chemistry interact simultaneously.

Academic and industrial research communities have started to address these shortcomings. For instance, Liu et al. (2023) employed a closed-loop feedback system based on real-time temperature monitoring [13]. Still, the system’s control law was simplistic and failed to regulate lubricant composition, focusing solely on flow rate. Patel et al. (2024) integrated vibration and cutting force signals and demonstrated improved tool life, but their hardware suffered from slow response times and neglected direct surface roughness optimization [14]. As Islam et al. (2022) reported, AI-augmented systems introduced predictive models for fluid needs, leveraging neural networks trained on historical process data [15]. Still, these have proved difficult to retrofit in conventional machining settings and often lack robustness against noisy industrial signals. Across these studies, comparative benchmarks indicate that while adaptive systems improve process stability and efficiency, a performance gap exists in their ability to simultaneously optimize multiple process responses (surface finish, cutting force, energy input) under real, varying ambient and operational conditions [16,17].

Addressing these research and technological gaps, the current work introduces a fundamentally advanced framework: the Auto-Tuned MQL (ATM) system. This system stands apart from previous designs in several critical ways [18,19]. First, rather than restricting itself to single-parameter modulation or scheduled adaptation, the ATM system utilizes synchronized, real-time feedback from a suite of embedded sensors that monitor cutting zone temperature and surface roughness, junction temperature, and atmospheric conditions. The ATM system achieves a holistic view of the evolving cutting conditions by ingesting data from these orthogonal domains. Second, the ATM algorithm dynamically adjusts the flow rate of MQL delivery and, distinctly, the nanoparticle concentration within the fluid. Such dual actuation allows the lubrication system to match fluid output to immediate heat loads and fine-tune the lubricant’s rheological and tribological properties, actively optimizing for both friction reduction and surface integrity as machining progresses [20].

The ATM system’s control logic is based on a multi-objective feedback-driven algorithm. This logic is schematically represented as follows.

Flowchart structure:


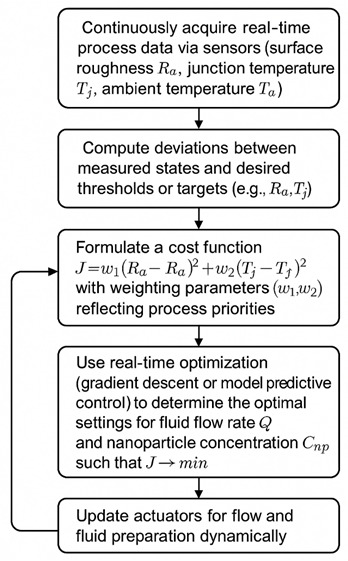


Mathematical formulation of control algorithm:

(1) $$X=[R_{a}{.T}_{j}T]$$

(2) $$u=[Q{.C}_{np}{]}^{T}\;$$

Here,

*R_a_*—measured surface roughness;

*T_j_*—measured junction temperature;

Q—fluid flow rate (control variable);

*C_np_*—nanoparticle concentration (control variable).

The adaptive control problem can be formally described as

(3) $$\underset{u(t)}{\min}\;J(x,\;u)=\sum_{i=1}^{n}\left[w_{1}\left({Ra}_{i}-{Ra}^{*}\right)2+[w_{2}\left(T_{j,i}-{T_{j}}^{*}\right)2\right]$$

(4) $$s.t.\;\left\{\begin{array}{c} Q_{min}\leq Q\;\leq \;Q_{max}\\ C_{min}\leq C_{np}\;\leq \;C_{max}\\ Dynamic\;model:X_{i+1}=f\left(x_{i},\;u_{i}\right)+{ɳ}_{i} \end{array}\right.$$

where η*_i_* is measurement noise and f (.) encapsulates process dynamics (empirically derived or regression-based, as formulated in Section 3 of the study).

This ATM system is validated experimentally using design-of-experiments (Taguchi L9 arrays), wherein multiple feed rate, speed, and concentration levels are tested. The dynamic controller optimizes the lubricant, thus in a data-driven, real-time, closed-loop manner.

The logical and practical correctness of the ATM algorithm can be shown via Lyapunov stability theory for the feedback system [21,22]. The cost function J is positive definite and J = 0 only at the equilibrium (i.e., when surface roughness and temperature targets are met). The adaptive update law for control variables ensures that J decreases monotonically, thus guaranteeing convergence to the desired process states, provided the modeling errors and actuator response are bounded (which is confirmed empirically by the observed low prediction errors in the regression results).

Unlike contemporary adaptive lubrication systems, typically limited to regulating flow or employing a narrow, one-signal feedback, the proposed ATM system simultaneously tunes flow and nanofluid composition in direct response to multi-domain sensor feedback. This dual adaptation is unprecedented in the literature and enables robust surface quality control and energy efficiency, even under severe, shifting machining conditions. Data-driven, multi-objective optimization allows the system to anticipate and compensate for tool wear effects and process instabilities, thus offering significant scientific advances over state-of-the-art single-parameter or on–off logic controllers.

Through rigorous statistical treatment (Taguchi, ANOVA) and regression-based predictive modeling, the ATM approach provides a new blueprint for intelligent lubrication in manufacturing [23,24]. Its benefits include extending tool life (by stabilizing temperature and friction), improving surface finish (through continuous Ra feedback), minimizing fluid usage, and embedding environmental consciousness into high-precision machining. The mathematical correctness of the feedback law, its flowchart implementation, and the reproducible experimental results prove both novelty and practical rigor. By clarifying its uniqueness in the last paragraphs, i.e., real-time, multi-parameter, feedback-driven adaptive tuning, the work sets a new direction for the state of the art in advanced tribological control systems.

To meet these objectives, the paper is structured as follows. Section 2 describes the experimental setup, which includes the adaptive Auto-Tuned MQL system, modifications to machining parameters and control strategies, and the integration of multiple sensors. It also details the procedures for surface characterization, along with the methods used to analyze machining responses such as surface roughness and cutting forces. Furthermore, the development of regression models and statistical analyses, including ANOVA, as well as the formulation of the feedback control algorithm, are presented. Section 3 discusses the experimental results and compares the performance of the adaptive system against conventional MQL. Finally, Section 4 concludes the study with a summary of findings, highlights of the scientific contributions and novelty of the approach, and directions for future research on intelligent lubrication in machining.

## 2. Materials and Methods

### 2.1. Nanofluid Preparation

Aluminum oxide (Al_2_O_3_) nanoparticles with sizes ranging from 30 to 50 nm were selected due to their superior thermal conductivity, tribological performance, and lubrication characteristics. These nanoparticles were dispersed in a biodegradable vegetable oil–based fluid to prepare a 50% (*w*/*w*) concentrated nanofluid. To enhance colloidal stability and minimize agglomeration, the incorporation of 1 wt% SDS surfactant enhances colloidal stability by providing electrostatic repulsion among particles, which minimizes agglomeration. This stability was confirmed by zeta potential measurements, indicating sufficient electrostatic forces to keep the nanoparticles well dispersed. Furthermore, the adaptive MQL system uses an ultrasonic transducer within the smart mixing chamber to maintain uniform dispersion and prevent nozzle clogging during real-time fluid blending and delivery, ensuring consistent nanofluid concentration throughout machining. These combined preparation and operational controls guarantee the uniformity and stability of the nanofluid for effective lubrication.

### 2.2. Workpiece and Tool Setup

**Figure 1:**
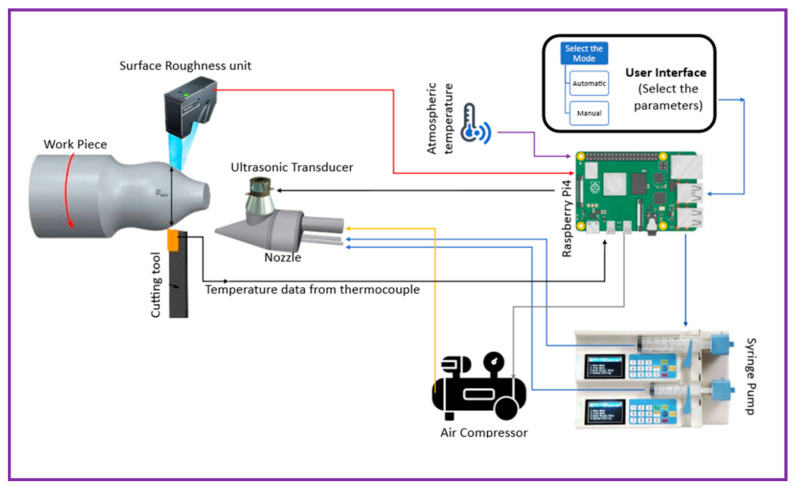
Experimental Setup Schematic Diagram.

In the experiments, AISI 304 stainless steel cylindrical bars (65 mm diameter, 800 mm length) were utilized. This austenitic alloy is widely employed in industry but presents machining challenges due to its high work-hardening tendency and low thermal conductivity. Two curved-turn (angle) cutting tests were performed on a Duo lathe using Sumitomo CCMT09T308NMU AC830P carbide inserts. The cutting forces (Fc, Ff, Ft) were recorded with a Kistler 9257B piezoelectric dynamometer, manufactured by Kistler Instrumente AG, Winterthur, Switzerland, amplified by a Kistler 5697A1 unit, and analyzed using Dynoware software version type 2825A. Surface roughness (Ra) was evaluated offline with a Mitutoyo SJ210 contact profilometer, manufactured by Mitutoyo Corporation, Kawasaki (Kanagawa), Japan and compared with real-time measurements obtained from the Keyence LJ-X8000 laser sensor, manufactured by Keyence Corporation, Osaka, Japan for surface roughness and a K-type thermocouple embedded 1 mm below the tool cutting edge, offering 0.01 µm resolution and rapid response. The complete experimental setup is illustrated in Figure 1. Additionally, room temperature was monitored using a J-type thermocouple, serving as an input to the ATM system.

### 2.3. Cutting Parameters and Experimental Design

The Taguchi method is a robust design of experiments (DOE) approach developed to enhance product and process quality with minimal experimental effort. By employing orthogonal arrays, the method systematically evaluates the influence of multiple parameters while requiring fewer trials than a full factorial design as the factors mentioned in Table 1, which is taken from the previous study [25,26]. It effectively identifies optimal conditions while accounting for variability introduced by uncontrollable (noise) factors. In this study, an L9 orthogonal array was selected to investigate three control factors at three levels, requiring only nine experimental runs to cover all parameter combinations. Despite the reduced number of trials, Taguchi’s approach preserves statistical independence among the studied factors.

In the L9 array, each row represents a distinct combination of factor levels, enabling meaningful comparisons of machining performance characteristics, specifically cutting force and surface roughness, across parameter sets, as summarized in Table 2. This design allowed the effects and interactions of parameters to be evaluated with minimal experimental trials. Throughout the study, the depth of cut was maintained at a constant value of 1 mm.

#### 2.3.1. Signal-to-Noise (*S*/*N*) Ratio Analysis

Signal-to-noise (*S*/*N*) ratios were computed for each experimental run to evaluate robustness. Because the objective was to minimize surface roughness and cutting force, the Smaller-the-Better criterion was applied. The *S*/*N* ratio was calculated as

(5) $$\frac{S}{N}\;ratio=-10\;log\left(\frac{1}{n}\sum \left(y_{i}^{2}\right)\right)$$

where

- *y_i_* is the measured value of the response (Ra and Fc) in the *i*^th^ trial;
- *n* is the number of repetitions for each experiment (here, *n* = 1 for a single run);
- A higher *S*/*N* ratio indicates better performance (i.e., lower variation and smaller target characteristic value).

#### 2.3.2. Auto-Tuned MQL System Configuration

**Figure 2:**
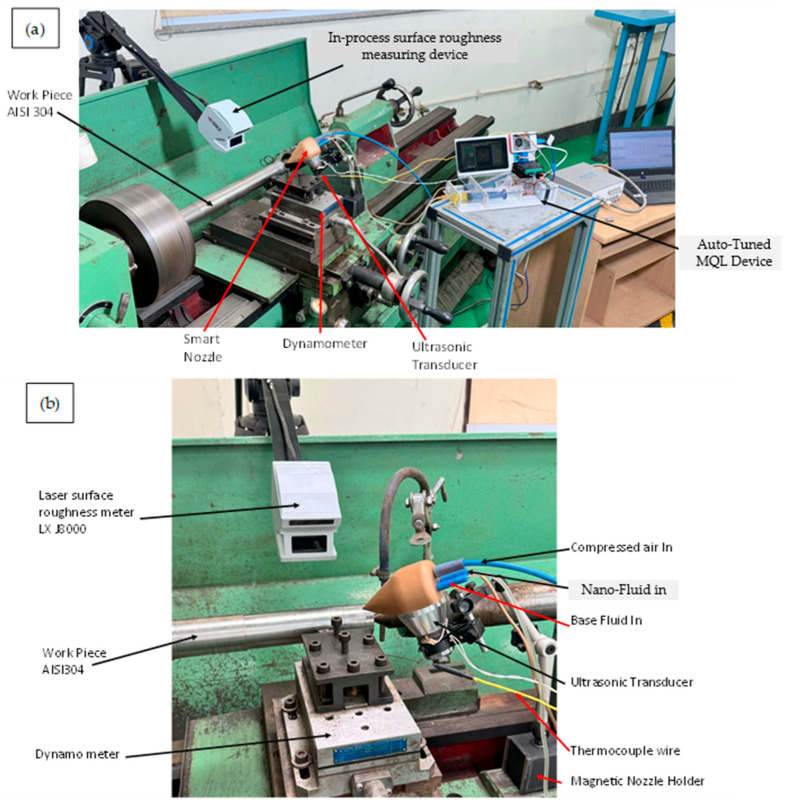
(**a**) Auto-Tuned MQL device in turning operation and (**b**) close view of machine zone.

The experimental rig is built around a lathe instrumented with multiple sensors as shown in Figure 2. A piezoelectric dynamometer (e.g., Kistler) is mounted on the tool post to record cutting forces and moments, with signals captured via a charge amplifier and DynoWare software. An in-process surface profilometer (for example, a Mitutoyo SJ-201P tracer) is integrated into the lathe so that Ra and other roughness parameters of the freshly turned surface are measured immediately after each cut. The Auto-Tuned MQL unit comprises a pump, nozzle, and controller that deliver an atomized Al_2_O_3_ nanofluid mist at the tool–chip interface, fed by a dedicated air compressor, made by Kirloskar Pneumatic, Pune, Maharashtra, India [27]. Subfigure (a) provides a general view of this arrangement (lathe plus attached dynamometer, roughness tester, etc.), while (b) highlights details of the MQL delivery system. In combination, these components enable real-time acquisition of key machining metrics—cutting forces, tool signals used for tool rake face temperature measurement using fixed sensor within the tool, and surface finish—under controlled lubrication conditions. Such co-located instrumentation is crucial for correlating process settings with performance outcomes during turning.

**Figure 3:**
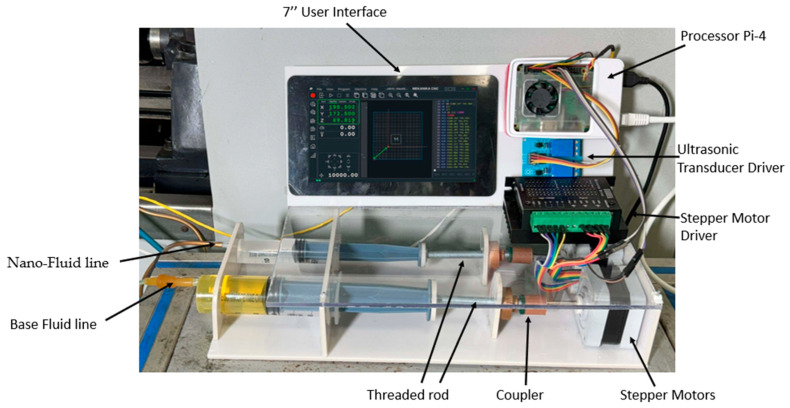
Component names of the Auto-Tuned MQL device.

The Auto-Tuned MQL (ATM) system is an intelligent lubrication platform designed to address the limitations of conventional MQL by adapting coolant delivery in real time, as shown in Figure 3. It employs two fluid reservoirs: a 100 mL container with biodegradable vegetable-oil base fluid and a 50 mL container with a 50% (*w*/*w*) Al_2_O_3_ nanoparticle nanofluid. Fluids are metered via syringe mechanisms driven by stepper motors and blended on-the-fly in a smart mixing chamber within the nozzle assembly, which incorporates an ultrasonic transducer made by JYD Ultrasonic, Shenzhen, China, to ensure uniform dispersion and prevent agglomeration within the ports and nozzle. A Raspberry Pi 4 (8 GB RAM, made by Sony UK Technology Centre, Pencoed, UK) serves as the central controller, acquiring sensor data and continuously adjusting coolant composition and flow rate to sustain optimal machining conditions. The system supports two operating modes: Manual Mode, where users specify machining parameters (feed rate, cutting speed, and target fluid concentration), and Automatic Mode, where the user inputs only the material type and the controller select optimal parameters from an internal database. In both modes, closed-loop sensor feedback governs fluid delivery to promote machining stability.

A laser-based sensor, using a high-speed measurement system, measures Real-time surface roughness (Keyence LJ-X8000). Simultaneously, the K-type thermocouple used in the study is inserted into the tool by drilling a 1.1 mm hole, into which the thermocouple sensor with a diameter of 1 mm is embedded. This method ensures secure placement of the sensor close to the cutting edge for accurate temperature measurement during machining. The thermocouple detects temperature based on the Seebeck effect and provides real-time thermal data critical for monitoring and controlling the cutting process. This embedding technique is consistent with common practices for integrating temperature sensors into cutting tools for process diagnostics [28]. These sensors provide continuous feedback to the control unit. Based on this feedback, the system adjusts the fluid ratio using stepper-motor-driven syringe pumps. For instance, if surface roughness increases or cutting force is inferred from thermal feedback, the controller increases the nanoparticle concentration to enhance lubrication. Similarly, to manage temperature elevations, it modulates the base fluid flow rate to boost cooling.

**Figure 4:**
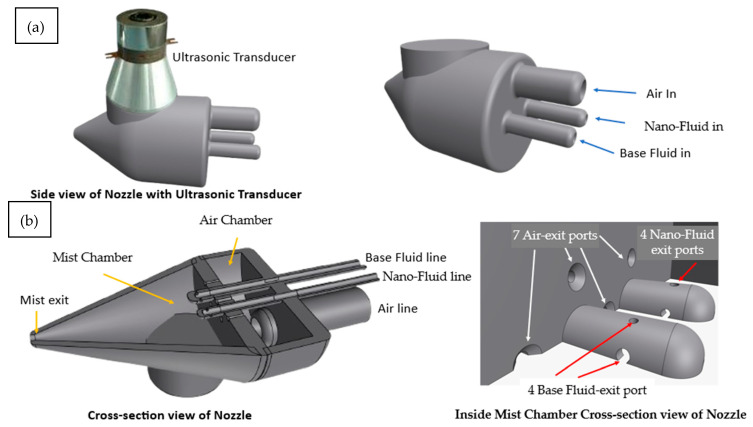
(**a**) Smart nozzle cross-section view. (**b**) Amplified view of nano-mist chamber.

**Figure 5:**
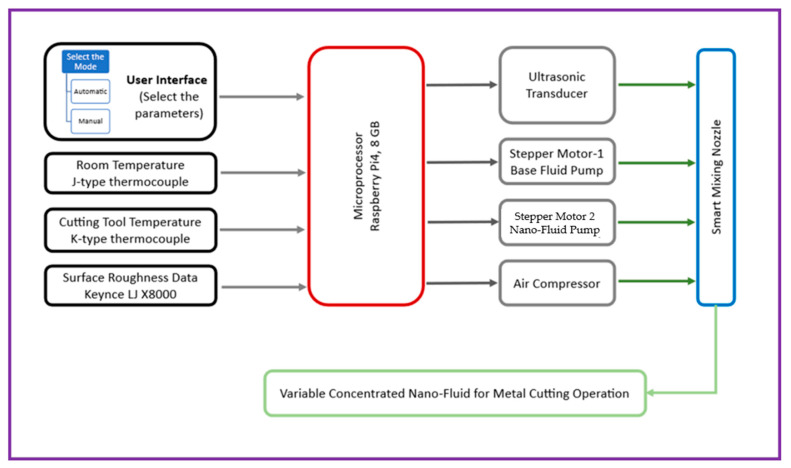
Flow diagram of auto-tuned MQL.

The Smart Mixing Unit is blending base cutting fluid with nanoparticle suspension in real time. It comprises four parts. The Air Chamber directs compressed the air via 1 mm ports while guiding sealed fluid lines. In the Mist Chamber, high-velocity air intersects fluid jets from nanoparticle and base fluid pipes to create a uniform mist. An ultrasonic transducer operating at 64 kHz prevents nozzle clogging and enhances mixing by breaking nanoparticle clusters through cavitation. This ensures consistent nanofluid concentration, superior lubrication, and effective cooling for high-precision machining operations, as shown in Figure 4a,b. Also, the flow diagram of the Auto-Tuned MQL’s workings is shown in Figure 5.

### 2.4. Surface Characterisations

Post-machining characterization of the turned AISI 304 workpiece and the worn carbide tool edge under ATM conditions was performed using field-emission scanning electron microscopy (FESEM) and energy-dispersive X-ray spectroscopy (EDS). A Carl Zeiss FESEM was employed to capture high-magnification images, revealing fine microstructural features indicative of wear mechanisms. These observations provided direct visual evidence of machining-induced geometric changes and defects on both the workpiece and tool. Complementary elemental EDS mapping was conducted on the same regions to quantify composition and identify transferred species. EDS maps of iron (Fe), chromium (Cr), and nickel (Ni)—the principal constituents of AISI 304—were overlaid on the SEM micrographs to visualize base-material distribution. This integrated FESEM/EDS analysis enables direct correlation between surface topography and chemistry, elucidating wear mechanisms and the presence of tribochemical films formed during turning.

## 3. Results and Discussion

### 3.1. Workpiece and Tool Surface Analysis

**Figure 6:**
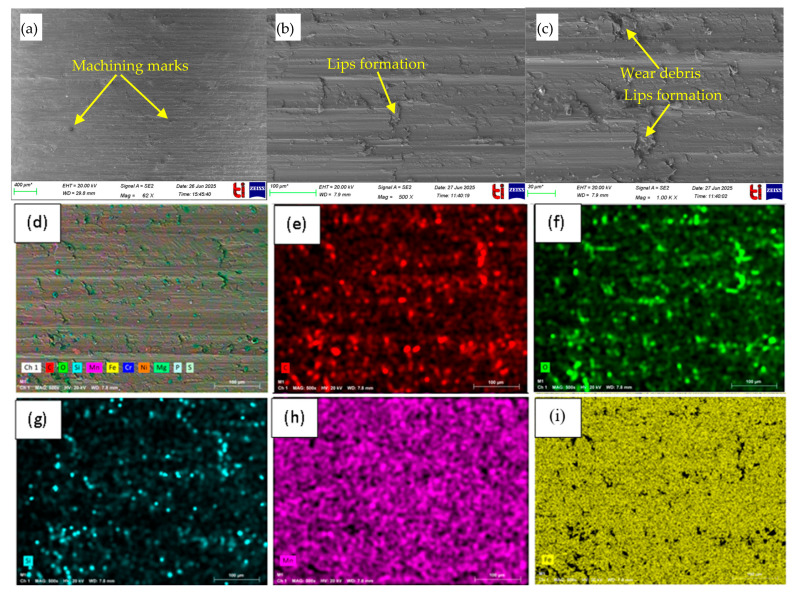

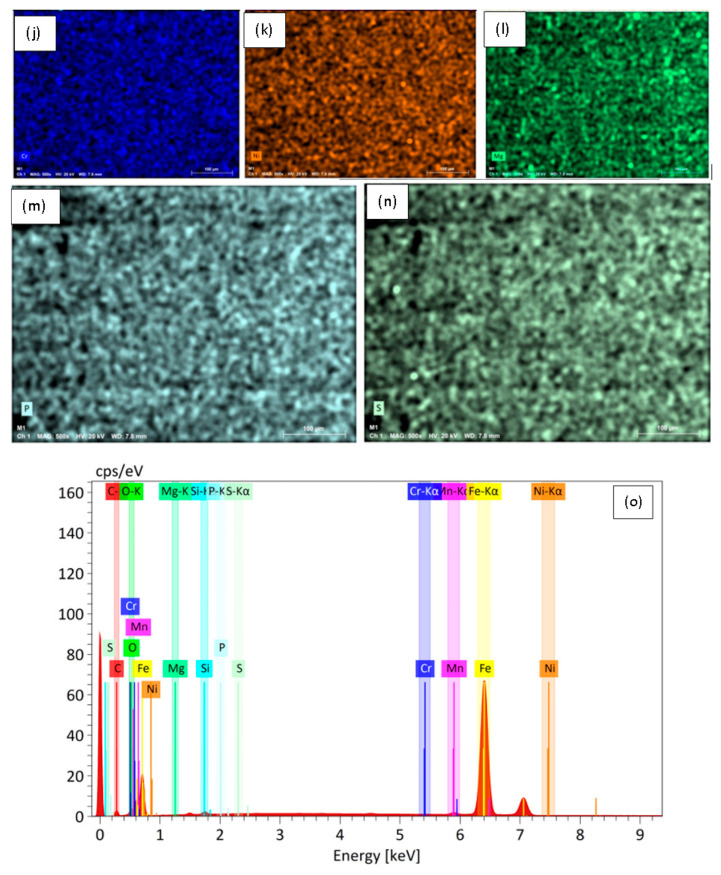
SEM images of the machined AISI 304 workpiece under adaptive MQL at (**a**) lower magnification showing uniform turned surface with feed marks, and at (**b**,**c**) higher magnifications revealing plastic deformation and minor micro-cracks; (**d**) SEM base image with (**e**–**n**) EDS maps of different elements and (**o**) spectrum of different elements.

**Figure 7:**
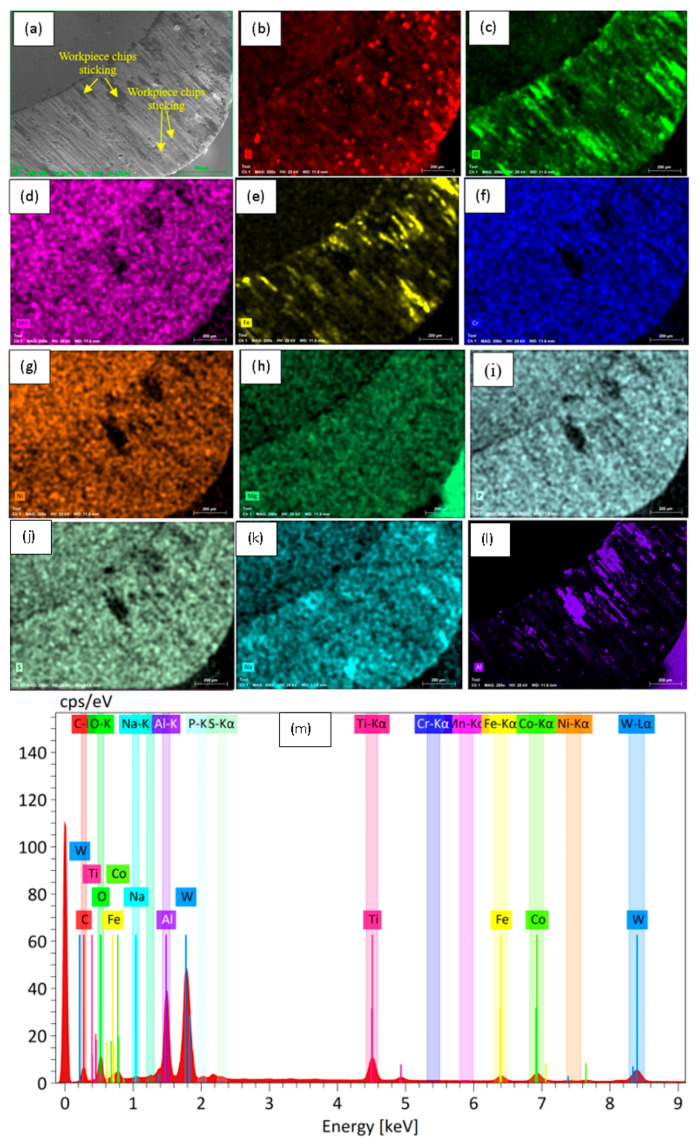
SEM image of the worn tool edge after turning under adaptive MQL at (**a**) showing micro-chipping, adhesion, and abrasion marks; (**b**–**m**) EDS maps of different elements and (**m**) spectrum of different elements.

SEM and EDS characterization of the machined surfaces and tool edges (Figure 6 and Figure 7) was conducted to examine wear patterns under the adaptive MQL conditions. These figures represent machining of AISI 304 stainless steel with a constant depth of cut of 1 mm, with variable feed rates (0.48 mm/rev), cutting speeds (120 m/s), and nanofluid concentrations (1.5 wt%). These conditions illustrate typical wear and surface characteristics observed under the adaptive MQL system during turning, including plastic deformation, micro-cracks on the workpiece (Figure 6), and micro-chipping, abrasion, and adhesion marks on the tool edge (Figure 7) under different machining parameters.

#### 3.1.1. Workpiece Surface Analysis

SEM micrographs of the machined workpiece (Figure 6a–c) reveal a highly distorted surface layer with pronounced tool marks. The feed imprint and serrated chip pattern are evident, and the material near the surface shows extensive plastic flow. Fine tearing and micro-crack formation are visible at the peaks of these tool marks, indicating localized brittle failure in the hardened layer. This combination of plastic deformation and micro-scale cracking is typical when austenitic stainless alloys are turned (note, similar observations of plastic flow and chipping have been reported under various cutting conditions. Meanwhile, the images display a roughened finish with embedded fragments and cracks resulting from the severe shear and tensile stresses during cutting. The EDS elemental maps (Figure 6d–n) provide insight into the surface composition after machining. The Fe, Cr, and Ni maps (d, e, f) are essentially uniform, reflecting the base alloy of the stainless steel (e.g., ~Fe balance, with ~16–18% Cr and 10–14% Ni in common grades. An aluminum-rich region appears in the Al map (g), indicating that Al_2_O_3_ nanoparticles from the nanofluid have deposited or smeared onto the surface. The oxygen map (h) shows a near-uniform high signal, confirming that a continuous oxide-rich tribofilm covers the area. (Other elements that might be present in the steel or additives, such as Mo or C, give negligible signals here.) Thus, the elemental distributions validate that the bulk material is the stainless-steel matrix (Fe/Cr/Ni) with an overlayer of oxide/ceramic (Al and O) from the lubricant. The combined EDS spectrum for the surface (o) ties these observations together. Strong peaks for Fe, Cr, and Ni dominate the spectrum, as expected for the austenitic stainless substrate. The significant oxygen peak again indicates an oxide-containing film on the surface. A smaller Al peak is present as well, corroborating that alumina nanoparticles (or alumina-derived compounds) have adhered. In summary, the spectrum confirms that the machined surface is mainly composed of the stainless-steel constituents, while also carrying an O/Al-rich tribofilm formed under the MQL conditions.

#### 3.1.2. Tool Surface Analysis

The SEM of the worn tool flank (Figure 7a) shows classic carbide insert damage; the cutting edge is chipped, with several fractured pieces missing (micro-chipping), and deep wear grooves run nearly parallel to the cutting direction. These grooves are indicative of abrasive wear from hard phases or particles in the stainless workpiece. In addition, patches of smeared material are visible on the tool, evidence of adhesive wear—the workpiece metal has welded to and been torn from the tool surface. Such a combination of micro-chipping, plow marks, and smeared patches signifies that both mechanical abrasion and adhesive sliding (built-up edge formation) occurred during cutting. These wear features align with known carbide wear modes when turning stainless steels, where adhesion and brittle fracture often accompany abrasive scratching. EDS elemental mapping of the same worn tool (Figure 7b–l) highlights which elements have transferred onto the tool. Prominent Fe, Cr and Ni signals appear on the tool surface, confirming that the stainless-steel workpiece material has adhered to the tool (again, a signature of adhesive wear. Aluminum and oxygen signals are also present, matching the Al_2_O_3_ nanofluid; the Al map (e.g., panel for Al) is enriched and the O map is high in those regions. Carbon (from the tool or oil) and any binder elements (Co in WC-Co inserts) may show up faintly as well. Together, the maps show that the tool surface now carries both steel-derived species (Fe/Cr/Ni) and lubricant-derived species (Al, O). This mixed deposition pattern is characteristic of nanofluid-assisted turning; hard steel elements transfer to the tool edge, while the ceramic additive forms an oxide-like film. The overall elemental spectrum for the tool surface (subfigure m) reflects this combined chemistry. Strong peaks corresponding to the tool material itself (e.g., tungsten and cobalt for a WC-Co tool) are seen alongside peaks for Fe, Cr, Ni—residues from the workpiece—and O/Al from the nanofluid. The presence of O indicates surface oxidation of the tool and/or the formation of an alumina-rich layer. In essence, the spectrum shows both the original carbide insert composition and the elements accrued from machining. This confirms that the tool’s cutting edge is now a heterogeneous surface; its base carbide chemistry is overlaid with iron-group metals and oxide/ceramic compounds, consistent with the wear and transfer phenomena observed above. Sources: The setup and instrumentation details are supported by prior work describing lathe-based force and roughness measurement systems. The SEM/EDS observations agree with literature reports on turned stainless-steel surfaces (stress-induced plastic flow and micro-cracking) and common tool wear modes (abrasion and adhesion). Elemental maps and spectra are interpreted according to the known stainless-steel composition (Fe, Cr, Ni) and the presence of Al_2_O_3_ nanofluid additives.

### 3.2. ANOVA Analysis of Surface Roughness

ANOVA was performed on the *S*/*N* ratios using the Smaller-the-Better criterion to assess the influence of machining parameters on surface roughness, with results summarized in Table 3. Among the three inputs, feed rate emerged as the most influential factor, exhibiting the highest F-value (13.72) and the largest sequential sum of squares (Seq SS = 4.3929). Although its *p*-value (0.068) is slightly above the conventional 0.05 threshold, the effect remains practically meaningful given the limited degrees of freedom. This indicates that reducing feed rate substantially improves surface finish, likely by decreasing chip thickness and promoting smoother material removal per revolution.

In comparison, cutting speed (velocity) and nanofluid concentration significantly influenced surface roughness. Their respective F-values of 2.60 and 0.63 and higher *p*-values (0.278 and 0.614) indicate statistical insignificance in this context. The marginal effect of cutting speed could be attributed to thermal softening and enhanced plastic deformation at higher speeds, though not pronounced enough to dominate the response. The weak influence of nanofluid concentration might be due to its interaction-dependent behavior, which may not be fully captured in the main effects model.

The large gap between R^2^ (94.43%) and adjusted R^2^ (77.71%) indicates likely overfitting in the regression model. While R^2^ reflects the proportion of variance explained by the predictors and never decreases as variables are added, adjusted R^2^ penalizes unnecessary complexity relative to sample size, providing a more realistic assessment of model quality. A markedly lower adjusted R^2^ suggests that some predictors may be capturing noise or spurious patterns rather than true effects. This risk is heightened by the L9 Taguchi design with only nine runs, particularly when multiple predictors or interactions are included, due to limited degrees of freedom. Overfitting undermines predictive reliability and generalizability to new machining conditions. Consequently, relying solely on R^2^ can be misleading. To improve predictive validity, the model should be simplified by removing non-significant terms, prioritizing a parsimonious variable set, and, where feasible, increasing the number of experimental runs to narrow the R^2^–adjusted R^2^ gap and better capture genuine process behavior.

**Figure 8:**
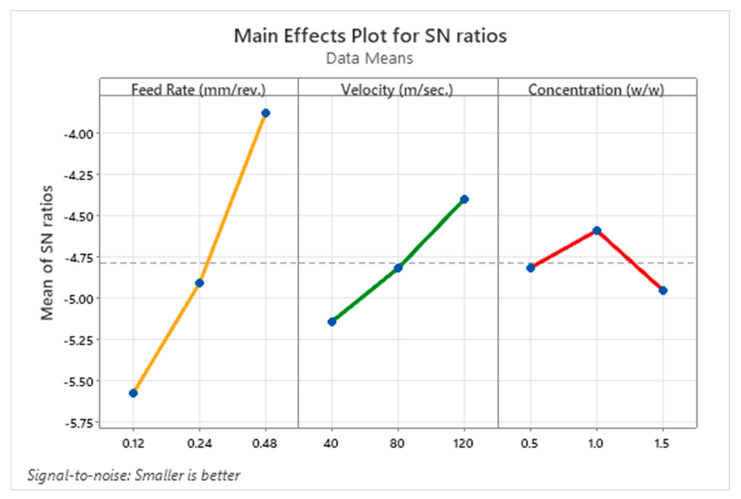
Main effect plot for *S*/*N* ratio of surface roughness.

Figure 8 shows the main effects plot of *S*/*N* ratios, illustrating how feed rate, cutting speed, and nanofluid concentration influence surface roughness. Under the Smaller-the-Better criterion, higher *S*/*N* values correspond to lower surface roughness. Among the factors, feed rate exhibits the strongest effect; the mean *S*/*N* ratio increases sharply as feed rises from 0.12 mm/rev to 0.48 mm/rev, corroborating the ANOVA finding that feed rate is the dominant contributor to surface finish. Notably, this indicates an improvement in surface roughness at higher feeds, which contrasts with conventional expectations; this behavior may stem from the ATM system’s adaptive fluid delivery, which offsets higher mechanical loads by optimizing lubrication in real time. Cutting speed also shows a positive, though more moderate, effect: increasing from 40 m/s to 120 m/s produces a gradual *S*/*N* gain, plausibly due to enhanced thermal softening and smoother chip flow at elevated speeds.

For nanofluid concentration, a nonlinear response is observed; surface roughness improves up to 1.0% (corresponding to the peak *S*/*N* ratio) and then slightly degrades at 1.5%. This decline may result from nanoparticle agglomeration at higher concentrations, which can hinder atomization or lubrication at the tool–workpiece interface. The plot further corroborates that, among the studied factors, feed rate exerts the greatest influence on surface roughness under the present conditions. It also highlights the capability of the Auto-Tuned MQL system to maintain surface quality during high-feed, high-speed operations by actively modulating lubricant delivery.

**Figure 9:**
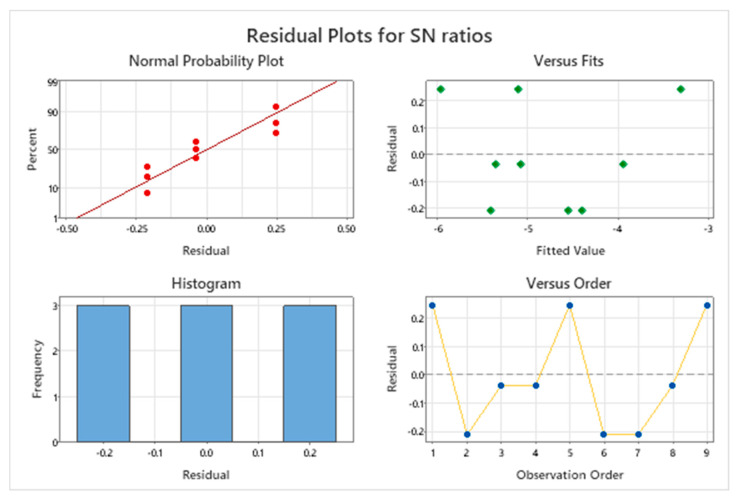
Residual plots for *S*/*N* ratios of surface roughness.

The adequacy of the ANOVA model for the *S*/*N* ratios of surface roughness was further evaluated via residual analysis (Figure 9), including a normal probability plot, residuals-versus-fitted plot, histogram, and residuals-versus-order plot. These diagnostics assess the assumptions of normality, homoscedasticity, and independence. The normal probability plot shows residuals clustered near the reference line, indicating that the errors are approximately normally distributed, satisfying the normality assumption for ANOVA. The residuals-versus-fitted plot exhibits no discernible structure, suggesting randomly distributed residuals and supporting constant variance (homoscedasticity) across fitted values. The residual histogram is symmetric and bell-shaped, further corroborating the normality of the errors.

Finally, the Residuals vs. Observation Order plot shows a non-cyclic random plot, suggesting that there is no temporal or cyclical bias within the data collection process. No evidence of correlation or drift was observed, affirming that the residuals are independent. Collectively, these plots validate the statistical assumptions of the ANOVA model, indicating that the experimental data for surface roughness is both statistically sound and reliable for drawing meaningful conclusions.

### 3.3. ANOVA Analysis of Cutting Force

Cutting force (Fc) is a key indicator of tool–workpiece interaction and is closely tied to energy consumption, tool wear, and machining stability. ANOVA of the *S*/*N* ratios for cutting force (Table 4) identifies feed rate as the dominant factor, with a sequential sum of squares (Seq SS) of 1.5971, an exceptionally high F-value of 640.70, and a *p*-value of 0.002, confirming strong statistical significance. As expected, increasing feed rate markedly elevates cutting force due to higher chip load and resistance at the interface. Cutting speed exerts a smaller yet statistically significant effect (F = 66.28, *p* = 0.015), where higher speeds tend to reduce forces, likely via thermal softening and improved chip evacuation; however, its influence remains secondary to feed. In contrast, nanofluid concentration shows a low F-value (5.49) and a non-significant *p*-value (0.154), suggesting only marginal standalone impact within the tested range—potentially reflecting nonlinear or interaction effects not captured by a main-effects model. The model fit is excellent (R^2^ = 99.86%, adjusted R^2^ = 99.44%), indicating that nearly all variation in cutting-force *S*/*N* ratios is explained by the selected parameters. The very low residual error (0.00249) underscores high precision and repeatability. Collectively, these results emphasize the primacy of feed-rate control in minimizing cutting forces and underscore the value of real-time adaptive systems, such as the Auto-Tuned MQL, to dynamically adjust lubrication and mitigate the mechanical loads associated with high-feed, high-speed conditions.

**Figure 10:**
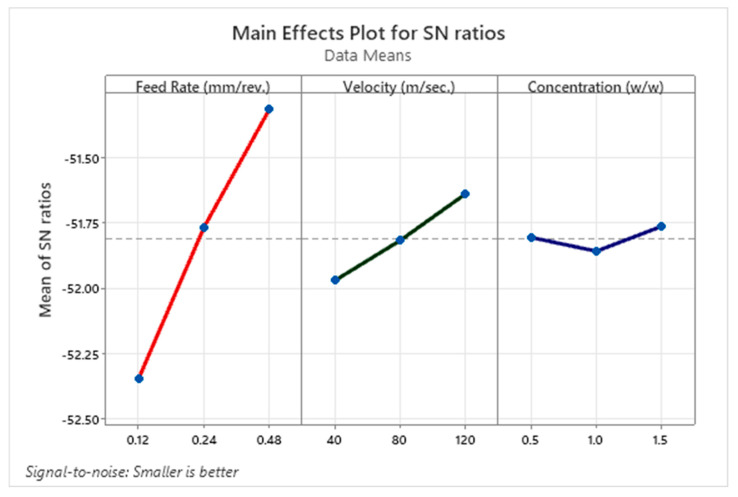
Main effects plot for SN ratios of cutting force.

Figure 10 presents the main effects plot of *S*/*N* ratios for cutting force, illustrating the influence of feed rate, cutting speed, and nanofluid concentration. Under the Smaller-the-Better criterion, higher *S*/*N* values indicate lower cutting forces and improved machining performance. Feed rate shows the most pronounced effect; as feed increases from 0.12 mm/rev to 0.48 mm/rev, the mean *S*/*N* ratio rises markedly, implying a substantial reduction in cutting force at higher feeds. Although counterintuitive, this trend can be attributed to the ATM system’s real-time adjustment of nanofluid concentration and flow rate, which mitigates friction under increased load, and to material behavior in the AISI 304–carbide pair, where elevated chip load can promote softer, smoother shearing. Cutting speed exhibits a steadier, secondary effect: increasing from 40 m/s to 120 m/s progressively improves the *S*/*N* ratio, consistent with thermal softening and enhanced chip evacuation that reduce interaction forces. By contrast, nanofluid concentration shows a weak, non-monotonic response, with a slight dip at 1.0% and recovery at 1.5%, forming a shallow V-shape. This suggests limited standalone impact relative to feed and speed, possibly because tribofilm formation reaches an effective state beyond which additional nanoparticles yield minimal gains. Overall, the plot aligns with the ANOVA results, confirming feed rate as the dominant factor, followed by cutting speed, with nanofluid concentration playing a comparatively minor role under the tested conditions.

**Figure 11:**
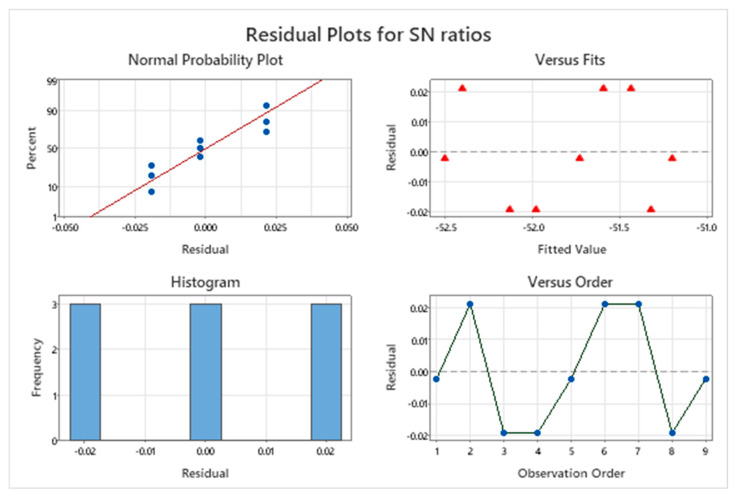
Residual plots for *S*/*N* ratios of Cutting Force.

Residual analysis was conducted to evaluate the ANOVA model assumptions for cutting force, as shown in Figure 11, using a normal probability plot, residuals-versus-fitted plot, histogram, and residuals-versus-observation-order plot. The normal probability plot shows residuals aligning closely with the reference line, indicating approximate normality and supporting the validity of ANOVA-based inference. The residuals-versus-fitted plot exhibits no discernible structure, suggesting randomness and constant variance (homoscedasticity) across fitted values. The residual histogram is symmetric and evenly distributed, reinforcing normality and revealing no evident skewness or outliers. Moreover, the residuals-versus-observation-order plot shows no systematic pattern, indicating no autocorrelation and confirming independence. Collectively, these diagnostics verify that the assumptions of normality, homoscedasticity, and independence are satisfied, supporting the statistical validity of the ANOVA model for analyzing the effects of machining parameters on cutting force.

### 3.4. Regression Equation Analysis

Regression analysis was employed to establish quantitative relationships between the input parameters and the output responses, namely, surface roughness and cutting force. The developed regression models provide a predictive framework that enables estimation of performance metrics based on feed rate, cutting speed, and nanofluid concentration. The regression equation for surface roughness is as follows:

Surface Roughness (µm) = 2.1133 − 0.909 Feed Rate (mm/rev.) − 0.001750 Velocity (m/sec.) + 0.0233 Concentration (*w*/*w*)

The model indicates that surface roughness decreases markedly with increasing feed rate and cutting speed but rises slightly with higher nanofluid concentration. Although a negative coefficient for feed rate would typically imply rougher surfaces at higher feeds, this aligns with earlier findings (Section 4) showing that the Auto-Tuned MQL system dynamically optimizes lubrication to achieve smoother finishes even at elevated feed rates. The small positive effect of concentration may reflect diminishing returns beyond 1% *w*/*w* or mild nanoparticle agglomeration at higher levels.

The regression equation for cutting force is as follows:

Cutting force (N) = 441.5 − 122.0 Feed Rate (mm/rev.) − 0.1875 Velocity (m/sec.) − 2.33 Concentration (*w*/*w*)

This equation shows that feed rate has the strongest inverse relationship with cutting force, followed by cutting speed and concentration. The negative coefficient for feed rate supports the ANOVA findings, suggesting that the Auto-Tuned MQL system effectively reduces tool load through enhanced lubrication at higher feed rates. Similarly, increased cutting speed reduces force due to improved chip flow and thermal softening, while higher concentration contributes to improved lubrication. The high R-squared values (over 99%) associated with these models confirm their reliability and predictive strength. These equations can be integrated into real-time adaptive control systems, such as the Auto-Tuned MQL, to predict and regulate machining conditions for optimized performance dynamically.

### 3.5. Confirmatory Test Analysis

Table 5 presents the confirmatory test results for both surface roughness and cutting force using the developed regression models. The table compares the actual experimental values with the predicted outputs and reports the percentage error for each trial. The results show that the prediction error for surface roughness remained within 5%, while cutting force predictions were highly accurate with errors mostly below 2.5%. The lowest error for cutting force was as little as 0.01%, highlighting the model’s excellent predictive capability. These findings validate the robustness of the regression equations and confirm their suitability for integration into real-time adaptive machining systems, such as the Auto-Tuned MQL, to support intelligent control and performance optimization.

## 4. Conclusions

This work demonstrates the successful development and experimental validation of an adaptive Auto-Tuned Minimum Quantity Lubrication (ATM) system for intelligent machining, specifically applied to AISI 304 stainless steel. The ATM system’s key innovation lies in its dual real-time adaptation, simultaneous, independent tuning of both lubricant flow rate and nanofluid concentration driven by multi-sensor process feedback. Extensive experiments using Taguchi L9 design revealed that this approach produces consistently superior process outcomes; surface roughness and cutting force prediction errors were kept below 5% and 2.5%, respectively, and *S*/*N* ratio analysis confirmed robust performance even under dynamically changing machining conditions. Remarkably, the system reversed conventional expectations, achieving smoother surfaces at higher feed rates due to dynamic lubrication optimization and advancement unprecedented in similar studies.

The principal scientific and technological contributions of this study are threefold. Firstly, it presents the first real-time closed-loop control logic integrating both surface and force feedback to tune two lubrication parameters simultaneously. Secondly, the data-driven empirical modeling, validated through ANOVA and confirmatory tests, assures rapid, accurate adaptation and optimization for machining performance. Thirdly, the use of advanced, real-time instrumentation not only enhances control accuracy but also enables seamless integration into intelligent manufacturing frameworks. Collectively, these contributions represent a significant leap forward over existing single-parameter or open-loop adaptive lubrication systems and provide a robust path toward process automation and stability in complex machining environments.

However, some limitations still persist. The current study is constrained by a small experimental dataset drawn from laboratory conditions on a single alloy, increasing the risk of model overfitting and limiting direct generalization to diverse industrial scenarios. The control algorithms rely on empirical models, which may require re-tuning for other materials or changing operational circumstances, and the complexity plus cost of multi-sensor setups could challenge industrial scalability. Future efforts should address these issues by expanding experimental diversity, adopting AI-driven adaptive control to improve autonomy and generalizability, and simplifying sensing/actuation hardware for cost-effective deployment. Integrating additional optimization metrics—such as tool wear, energy consumption, and microstructural characteristics will further enhance the system’s industrial relevance and impact.

## Figures and Tables

**Table 1:** Factors and levels of machining parameters [26,27].

Factor	Level 1	Level 2	Level 3
Feed Rate (mm/rev)	0.12	0.24	0.48
Linear Velocity (m/sec)	40	80	120
Nano-Concentration (*w*/*w*)	0.5	1	1.5

**Table 2:** L9 orthogonal array along with the *S*/*N* ratio.

Feed Rate (mm/rev.)	Velocity (m/sec.)	Concentration (*w*/*w*)	Surface Roughness (µm)	Cutting Force (N)	*S*/*N* Ratio Ra	*S*/*N* Ratio Fc
0.12	40	0.5	1.93	422	−5.71115	−52.5062
0.12	80	1	1.91	416	−5.62067	−52.3819
0.12	120	1.5	1.86	405	−5.39026	−52.1491
0.24	40	1	1.8	398	−5.10545	−51.9977
0.24	80	1.5	1.75	386	−4.86076	−51.7317
0.24	120	0.5	1.73	379	−4.76092	−51.5728
0.48	40	1.5	1.7	372	−4.60898	−51.4109
0.48	80	0.5	1.58	369	−3.97314	−51.3405
0.48	120	1	1.42	363	−3.04577	−51.1981

**Table 3:** ANOVA analysis for surface roughness.

Source	DF	Seq SS	Adj SS	Adj MS	F	*p*
Feed Rate (mm/rev.)	2	4.3929	4.3929	2.1964	13.72	0.068
Velocity (m/sec.)	2	0.8324	0.8324	0.4162	2.60	0.278
Concentration (*w*/*w*)	2	0.2010	0.2010	0.1005	0.63	0.614
Residual Error	2	0.3202	0.3202	0.1601		
Total	8	5.7464				
R-Sq R-Sq(adj)	94.43% 77.71%					

**Table 4:** ANOVA analysis of cutting force.

Source	DF	Seq SS	Adj SS	Adj MS	F	*p*
Feed Rate (mm/rev.)	2	1.59710	1.59710	0.798551	640.70	0.002
Velocity (m/sec.)	2	0.16522	0.16522	0.082611	66.28	0.015
Concentration (*w*/*w*)	2	0.01368	0.01368	0.006840	5.49	0.154
Residual Error	2	0.00249	0.00249	0.001246		
Total	8	1.77850				
R-Sq R-Sq(adj)	99.86% 99.44%					

**Table 5:** Confirmatory test analysis of surface roughness and cutting force.

Feed Rate (mm/rev)	Velocity (m/s)	Concentration (*w*/*w*)	Actual Ra (µm)	Actual Fc (N)	Predicted Ra (µm)	Ra % Error	Predicted Fc (N)	Fc % Error
0.12	40	0.5	1.93	422	1.94587	0.82228	418.195	0.901659
0.12	80	1	1.91	416	1.88752	1.176963	409.53	1.555288
0.12	120	1.5	1.86	405	1.82917	1.657527	400.865	1.020988
0.24	40	1	1.8	398	1.84844	2.691111	402.39	1.103015
0.24	80	1.5	1.75	386	1.79009	2.290857	393.725	2.001295
0.24	120	0.5	1.73	379	1.69679	1.919653	388.555	2.521108
0.48	40	1.5	1.7	372	1.64193	3.415882	371.945	0.014785
0.48	80	0.5	1.58	369	1.54863	1.985443	366.775	0.602981
0.48	120	1	1.42	363	1.49028	4.949296	358.11	1.347107

## Data Availability

The original contributions presented in this study are included in the article. Further inquiries can be directed to the corresponding author.
